# Urban governance to support adaptable solutions for conversion of residual street spaces into social spaces

**DOI:** 10.1038/s41598-025-16569-2

**Published:** 2025-09-01

**Authors:** Chiara Garau, Reza Askarizad, Francesco Pinna

**Affiliations:** 1 Department of Civil and Environmental Engineering and Architecture (DICAAR), University of Cagliari, Via Marengo 2, 09123 Cagliari, Italy; 2 Department of Urban and Regional Planning, Universidad Politécnica de Madrid, 28040 Madrid, Spain

**Keywords:** Residual street spaces, Leftover spaces, Social interaction, Castello historic neighbourhood, Space syntax, Socio-spatial equity, Environmental social sciences, Psychology and behaviour

## Abstract

Abandoned residual street spaces, frequently marginalised in formal urban planning frameworks, represent a significant yet unrealised resource for enhancing urban liveability, especially within the complexities of dense historic urban neighbourhoods. This study investigates how such underutilised spaces can be strategically transformed into vibrant social hubs. Focusing on the Castello neighbourhood in the historic centre of Cagliari, Italy, the research adopts a unique mixed-methods approach grounded in a sequential exploratory design. It begins with spatial analysis using space syntax techniques, including segment analysis, axial analysis, and visibility graph analysis, followed by GIS-based land use mapping and field-based qualitative assessments to identify and prioritise residual spaces based on their social potential. The findings highlight several high-potential sites characterised by pronounced spatial configuration and contextual qualities, suggesting practical insights into the design and governance of adaptive social spaces. The original contribution of this study lies in its incorporated and replicable methodological framework that combines quantitative spatial analysis with qualitative urban evaluation. The results not only advance the theoretical discourse on residual urban space but also provide pragmatic strategies for inclusive urban regeneration. The proposed framework is applicable across diverse urban contexts, offering a scalable model for cities aiming to enhance sociability through the adaptive reuse of neglected urban fragments.

## Introduction

As urban development progresses, the efficient use of space becomes increasingly important. Many cities contain residual street spaces, such as small, publicly accessible fragments of land including vacant lots, alley widenings, and under‑programmed corners, which lie outside formal land‑use designations and lack an assigned civic, commercial, or infrastructural function^1^. Although some of these pieces already accommodate informal everyday activities, they remain “leftover” from an urban governance perspective and are seldom targeted by systematic regeneration policies. Revitalising such residual spaces can improve urban life by addressing congestion, social disconnection, and inequitable access to public realm amenities^2^. Residual spaces, often referred to by various equivalent terms such as lost space, abandoned space, leftover space, informal space, brownfield, void, and wasted space, are typically considered indispensable latent opportunities for regeneration projects^3,4^. Many studies highlight the importance and potential of urban leftover spaces, or residual spaces, emphasising their role as a viable and sustainable adaptation strategy^5^. By repurposing existing land uses, infrastructure, and urban resources, these spaces offer a socially, economically, and environmentally sound solution for sustainable urban development^6^. In this study, the term “residual” does not imply complete abandonment but rather refers to publicly accessible urban spaces that, despite their formal designation as plazas or landmarks, remain under-programmed, lack urban design facilities that support inclusive social activity, governance support, or fail to fully realise their sociability potential. This classification is therefore context-sensitive and grounded in spatial performance and functional affordances and facilities rather than nominal land-use categories.

Despite their socio-ecological potential, many residual spaces in urban planning have been neglected and overlooked due to a lack of awareness about their possible benefits^7^. Residual land in dense urban areas provides open space that serves as a resting spot, facilitates social interactions, offers expansive landscape views, and establishes a visually productive connection to other parts of the city, often surpassing densely planted parks in these aspects^8^. Coleman^9^ categorised vacant lands as dead spaces, whereas Davidson and Dolnick^10^ defined them as lands not currently utilised for any purpose. Residual spaces are characterised by their uncertain nature in relation to other land uses, often appearing as voids rather than productive or functional spaces within the city^11^. The existing literature confirms that abandoned lands have the potential to reduce the sense of community, safety, and lead to neighbourhood decline, thereby impacting property values and the overall quality of life for residents^12,13^. Thus, the identification and classification of such leftover lands are crucial as they enable better reuse and regeneration of urban spaces, thereby facilitating effective social life among community members^14^.

To operationalise this concept in the context of Cagliari, a clear framework was developed to classify a space as ‘residual’ based on a combination of spatial, functional, and qualitative criteria. These included (1) morphological irregularities or fragmented forms, such as setbacks, recesses, and edge spaces; (2) functional underuse, including lack of ground-floor activity or active frontage; (3) syntactical underperformance, reflected by poor integration and connectivity; and (4) perceptual or experiential deficiencies, including absence of urban furniture, shading, or visual cues that support lingering and social presence. To do so, a framework that integrates syntactical underperformance (via Space Syntax), lack of functional programming (via QGIS), and qualitative deficiencies (via field assessments) was employed. This multidimensional approach ensures that residuality is not assumed merely from nominal designation, but is analytically and contextually derived. Therefore, what qualifies as a ‘residual’ space in this study is not only a leftover fragment but also a spatial node with untapped social potential, as revealed through both quantitative and experiential indicators.

In a time of increasing urbanisation and limited land resources, optimising the use of all available land is crucial, particularly in historic neighbourhoods where space is at a premium. Cagliari, a city rich in Italian history and culture, faces the challenge of managing residual street spaces scattered throughout its urban areas. Without defining structured methods to address these problems, such residual spaces may remain underutilised or relinquished because of their scattered locations. Implementing systematic approaches for Cagliari’s historic neighbourhoods can certify that leftover spaces are repurposed effectively and beneficially. An accurate analysis using socially revitalising techniques can suggest the required visions to make certain these spaces contribute positively to the urban environment and improve the social welfare of the inhabitants, thereby supporting executable urban governance. Converting underused spaces in historic districts into lively social centres can improve the quality of life for residents, providing new places for recreation, interaction, and community development. Thus, there is an urgent need for a systematic analysis to develop effective strategies for the adaptive reuse of urban voids in historic neighbourhoods that are considered hotspot tourist destinations. Such an analysis would facilitate the creation of sustainable urban governance that can transform these underutilised spaces into vibrant social hubs, thereby enhancing community well-being and preserving the cultural heritage of Cagliari.

This research aims to adopt an innovative procedure to transform residual street spaces into dynamic social areas. Establishing social spaces in urban areas is essential for improving community interaction and overall social well-being^15,16^. These spaces provide residents with gathering points, increasing walkability, enhancing their sense of belonging, and improving the overall quality of urban life^17,18^. This research emphasises the significance of converting residual street spaces into social spaces, recognising their potential to trigger social engagement and community development. Space Syntax is employed because its configurational rigorous attributes have been repeatedly validated as reliable predictors of pedestrian movement and social co‑presence in a wide range of urban settings^19,20^ thus providing an evidence‑based foundation for identifying street fragments most likely to support new social functions. Therefore, it is intended to conduct space syntax analysis as a primary method to identify the areas with the highest potential for social attractions for pedestrians. Following this, using on-site investigations, and adoption of geographical information system (GIS) maps, a set of strategies will be developed to transform residual street spaces into dynamic social areas. To achieve this, the following research questions are pursued:

1. How can space syntax analysis be utilised to identify residual street spaces with high potential for social interaction and pedestrian attraction?
2. What are the key factors in spatial analysis that indicate a space’s suitability for conversion of residual space into a social area?

Previous studies related to the identification and revitalisation of vacant lands have primarily focused on in-between spaces^21,22^, parking lots^23^, residual spaces beneath elevated highways^6^, social–ecological resilience in urban landscapes^11,24–26^, temporary uses of derelict urban spaces^27–29^, examination of theories on urban void spaces^4,8,30^, behaviour, cognitive and emotional patterns of users^31,32^, reuse and regeneration of residual spaces^14,33^, the association between vacant land and placemaking^34^, and the association between vacant lands and public health outcomes^2^.

However, the evolution of residual street spaces into dynamic social areas remains a growing topic of interest where further research is needed to enhance our understanding and diversify methodological approaches within the urban planning literature. This research intends to bridge this gap by not only identifying potential areas for social space conversion through space syntax analysis but also by developing adaptable strategies based on spatial analysis and on-site investigations. The innovative procedure proposed in this research will provide a unique framework for transforming overlooked urban spaces into vibrant social hubs, thereby advancing the discourse on urban regeneration and community development.

This study contributes to the growing field of urban regeneration by presenting a replicable, multi-scalar methodology that integrates syntactical analysis, GIS-based mapping, and qualitative field investigation for the identification and prioritisation of residual urban spaces within the historic fabric of Cagliari in Italy. While prior studies have often focused on temporary reuse or ecological rehabilitation, this research shifts the emphasis toward long-term sociability, inclusivity, and spatial equity in historic contexts. The originality of this work lies in its systematic combination of quantitative configurational metrics with qualitative assessments of experiential qualities, yielding a nuanced framework that bridges spatial performance with human-centred urban design in the historic neighbourhood of Castello. The proposed framework offers not only analytical precision but also practical relevance for governance-led urban transformation. Following this introduction, the paper progresses as follows: Sect. 2 details the methodology, including research design, data collection methods and analytical approaches. Section 3 presents the research findings and results, including spatial analyses and outcomes from field investigations. Section 4 discusses the implications of the findings, and suggesting future research directions. Section 5 concludes by summarising key findings, and reflecting on the study’s contributions.

## Materials and methods

This research employs a mixed-methods approach, combining both quantitative and qualitative methodologies to transform residual street spaces into dynamic social hubs. The research is organised into several phases, each concentrating on separate but interconnected analytical procedures. These methods comprise Space Syntax analysis, GIS mapping, and on-site investigations. This systematic approach ensures adaptive procedures into the spatial attributes of residual street spaces and their potential for social engagement. The research framework is designed to incorporate socio-spatial data to support the transformation of residual street spaces into social spaces. This study follows a sequential exploratory design, where spatial analysis using Space Syntax and GIS informs the subsequent stages of on-site investigations. The goal is to establish a clear link between spatial configurations and adaptive conversion of residual lands into sociable spaces, allowing for an adaptable urban governance strategy.

**Fig. 1:**
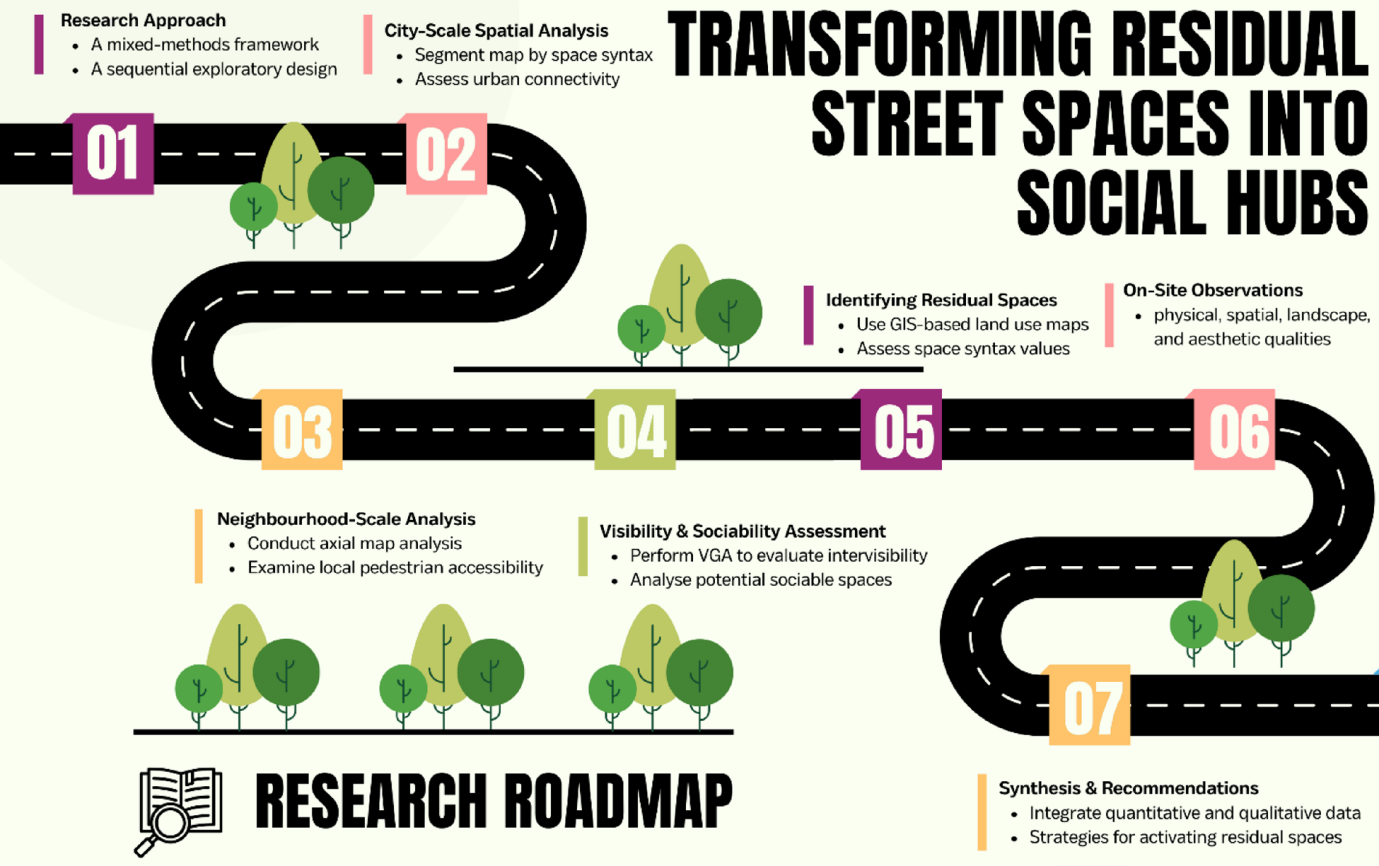
The roadmap illustrating the process of conducted research methodologies in this study.

To support broader applicability, this methodological framework is structured as a step-by-step process consisting of seven clearly defined phases, which are visualised in (Fig. 1). These include: (1) sequantial exploratory research approach, (2) city-scale spatial analysis using segment map, (3) local pedestrian accessibility using axial analysis, (4) intervisibility and sociability assessment using VGA, (5) residual space identification using GIS-based land use data, (6) incorporation of physical, spatial, landscape, and aesthetic qualities using field assessments, and (7) data synthesis and recommendations for socialising residual spaces. This sequence was designed for replication in other historic or morphologically complex cities, particularly where spatial fragmentation coexists with underused public assets.

### Spatial analysis using space syntax and QGIS

Space Syntax is a theory and set of analytical techniques that quantitatively describe the spatial configuration of the urban environment. Developed by Hillier and Hanson in the 1980s, it emphasises the relationship between space and social interactions, enabling researchers to predict movement patterns, accessibility, and the potential for social activities in different spatial layouts^35,36^. Building on the “natural movement” theory developed by Hillier et al.^19^ and their subsequent work^20^Space Syntax posits a mutual relationship in which spatial configuration both affords and is reinforced by pedestrian co‑presence, making configurational metrics reliable predictors of aggregate movement and social encounter.

In this research, Space Syntax functions as a key tool in evaluating the potential of residual spaces to be transformed into social spaces that attract and facilitate pedestrian activity. Space Syntax is particularly effective for this study’s objectives, as it offers a meticulous instrument to understand the configurational properties of urban space that promote or prevent social encounters. Through applying Space Syntax, this study aims to identify the most appropriate residual spaces with the highest potential to attract social activities, contributing to the broader goal of converting underutilised spaces into vibrant, and pedestrian-friendly areas.

The spatial analysis commenced with an overarching city-scale examination, using a segment map generated within the Space Syntax toolkit in QGIS 3.16. Segment map analysis divides the urban layout into interconnected street segments, quantifying spatial configuration to forecast movement patterns, which makes it a striking tool for examining city-scale accessibility^37^. The segment map was generated using road centreline data as the basis for constructing interconnected street segments within the urban fabric. The initial source of data is derived from the publicly accessible resource, such as Geoportal Sector of Cagliari Municipality, and Google Street Map, but was curated and adapted for research use. It should be noted that the dataset was cross-verified with satellite imagery and field surveys to ensure its spatial accuracy and completeness prior to analysis. After identifying the overall configurational status of the study area within the urban spatial network analysis, further analysis was carried out in the city centre using axial map analysis to examine the configurational properties of the target neighbourhood in more detail using UCL Depthmap Version 10.14.00b. This software was originally developed by Alasdair Turner at University College London^38^. Axial analysis is a well-established method in Space Syntax, where the city is broken down into a network of the longest and fewest straight lines that represent possible pedestrian routes^39^. This analysis allows for the evaluation of several syntactical values that are critical in understanding pedestrian movement.

Afterward, Visibility Graph Analysis (VGA) was performed within the territory of the studied neighbourhood to assess the conformity between visibility values and potential lands for the socialisation process. VGA is a specialised technique in Space Syntax that analyses the intervisibility between different points within a space^40^. By breaking down a space into a grid set of points, VGA creates a graph where the nodes represent these points and the edges represent lines of sight. Unlike axial analysis, which focuses on movement between spaces, VGA specifically analyses how visible different points within a space are to one another, thereby helping to understand how people perceive and interact with the environment. The aim of this analysis is to provide a set of quantitative assessments regarding the relationship between the numerical values of syntactic analysis and potential social hubs within the studied neighborhood.

In this process, the integration value reflects how well a street or space is linked to the wider urban network. Spaces with a higher integration value are more frequently used and easier to reach, indicating their importance in community life^41^. The connectivity metric evaluates the number of immediate connections a space has with other spaces. Spaces with higher connectivity are more likely to serve as accessible hubs for movement and activity^42^. The degree of spatial control, reflecting how a space regulates access to its neighbors, provides insights into its suitability for surveillance^43^. Additionally, the visual clustering coefficient helps pinpoint potential pausing spaces, as significant view changes are likely to attract resting pedestrians^44,45^. By performing axial analysis at the city scale and VGA at the neighbourhood scale, the available residual spaces in the historic centre were specifically highlighted as areas of interest, indicating their significant potential for pedestrian movement and social activity.

### Identification of residual spaces using QGIS

Once the syntactical analysis was complete, the next step was to focus on the identification of residual spaces, which are often leftover or underutilised areas in the urban fabric, such as vacant lots, alleyways, or spaces between buildings. Using GIS-based land use maps, these residual spaces were identified within the historic centre of Cagliari, where the highest syntactical values were observed. It should be noted that the base land use map was initially obtained from reliable sources^46^ after which an updated version of the land use data was created based on the research objectives and complemented by field investigations to identify potential residual lands in this study using QGIS 3.16 software. The potential of residual spaces to be transformed into lively public areas is often overlooked in urban planning; however, this can be achieved if their spatial configuration is designed to facilitate pedestrian activity. By combining the syntactical values obtained from the VGA with the GIS land use data, the potential of these residual spaces to attract social activities was systematically evaluated. The goal here was to ensure that these leftover spaces were not only physically available but also well-situated within the urban network to serve as social spaces, maximising their ability to facilitate pedestrian engagement.

To systematically prioritise the most influential urban spaces based on spatial qualities derived from Space Syntax analysis, a structured methodological approach was employed. This approach assigns a weighted ranking system to each syntactical variable—Integration, Connectivity, Control, and Clustering Coefficient—to quantify their relative significance in shaping urban movement and social interactions. Each location is ranked within its respective variable, with the highest value receiving 10 credits, the second highest 9 credits, and so on until the tenth rank, which receives 1 credit. By summing these credits across all four spatial metrics, a cumulative priority score is established for each space, allowing for a holistic assessment of its spatial prominence, with the results shown in a heatmap. By aggregating these syntactical values, the methodology highlights urban spaces with the highest cumulative potential for movement, interaction, and socio-spatial significance, ensuring that interventions focus on areas that maximise urban accessibility, connectivity, and usability.

This identification process did not rely solely on syntactical outputs; instead, residual spaces were filtered through a two-tiered classification system. The first tier was based on functional underutilisation, identified through GIS-based land use layers and confirmed by field observations. Specifically, spaces were considered “residual” if they lacked clearly defined civic, commercial, or residential functions—such as vacant lots, unprogrammed plazas, marginal alley widenings, or open areas without consistent land-use designation or pedestrian amenities. These spaces typically exhibited low levels of programmed activity, limited public infrastructure, and minimal formal integration into daily urban routines. The second tier then assessed these preliminarily identified spaces for their spatial performance using Space Syntax analysis (e.g., integration, connectivity, control, clustering coefficient). This hybrid approach ensured that the classification of residual spaces was grounded not only in observable functional voids within the land use structure, but also in their latent potential for social revitalisation, as inferred from their configurational characteristics and visibility within the urban grid.

### Field investigations & qualitative assessments

Subsequent to the syntactic and GIS-driven detection of leftover areas, on-site examinations were performed to more thoroughly assess the appropriateness of these locales. This phase was critical for confirming the outcomes from the numerical assessment and for judging the descriptive features of these spaces, which cannot invariably be ascertained through syntactic analysis alone. The research design associated with qualitative assessments through field investigations was developed based on a set of theoretical underpinnings, which were found to be influential according to the previous literature. Hence, factors such as physical characteristics, spatial qualities, landscape elements, and aesthetic qualities were examined during the field investigations. These field investigations were accomplished through direct observation, photography, and note-taking.

To ensure greater objectivity and minimise individual bias, a structured assessment rubric was developed and applied by a group of twelve local experts—urban planners and architects affiliated with the University of Cagliari—who are deeply familiar with the Castello neighbourhood. The number of participants was determined based on the principle of saturation^47,48^ ensuring sufficient diversity of expert input without redundancy. Each expert was provided with standardised datasheets and prior information on the spatial analysis results. Using a predefined 1–10 scale, they independently rated the residual spaces across three dimensions: physical characteristics, landscape features, and aesthetic appeal. It should be noted that the spatial analysis assessment was not scored by an expert viewpoint and was solely operationalised through the results obtained from syntactical analysis. Final scores were derived by averaging these expert assessments, providing a more balanced and informed prioritisation of the residual spaces.

Physical characteristics refer to the size, shape, and spatial arrangement of each residual space^49,50^ while spatial qualities reveal how the space relates to its surrounding built environment, including its level of visibility and accessibility as verified through syntactic analysis^51,52^. Additionally, the presence of greenery and picturesque views of the city, which are among the city’s remarkable attractions, were considered landscape elements in these investigations^53,54^. Finally, aesthetic qualities refer to the space’s overall visual appeal, which can significantly influence its usage by pedestrians^55–57^. Accordingly, the analogous procedure for systemtic prioritisation according to the assigned weighted ranking system was applied in order to generate a comprehensive profile assessment for each identified residual space. It should be noted that the results of the evaluations were visualised using a spectrum heatmap.

This combination of quantitative syntactical analysis and qualitative investigations provided a thorough assessment of the potential for each residual space to be transformed into a social space. By understanding both the configurational properties and the experiential qualities of these spaces, the study ensures that the proposed interventions will align with both pedestrian movement patterns and social behaviour, making them highly likely to succeed as urban governance tool in promoting social activities. To summarise, the use of Space Syntax in this study enables a deep comprehension of the spatial possibilities within leftover areas. By pinpointing significant spatial characteristics using VGA and confirming them through on-site observation, this analysis verifies that the chosen residual spaces are suitable both in terms of their spatial configuration and physical attributes for transformation into lively public spaces that encourage pedestrian flow and social interaction. This ensures that spatial metrics are interpreted through a contextual lens that captures morphological, perceptual, and experiential aspects of each space, aligning quantitative outputs as well as their social relevance. The process of conducting the research procedures is illustrated in (Fig. 1).

### Study area

**Fig. 2:**
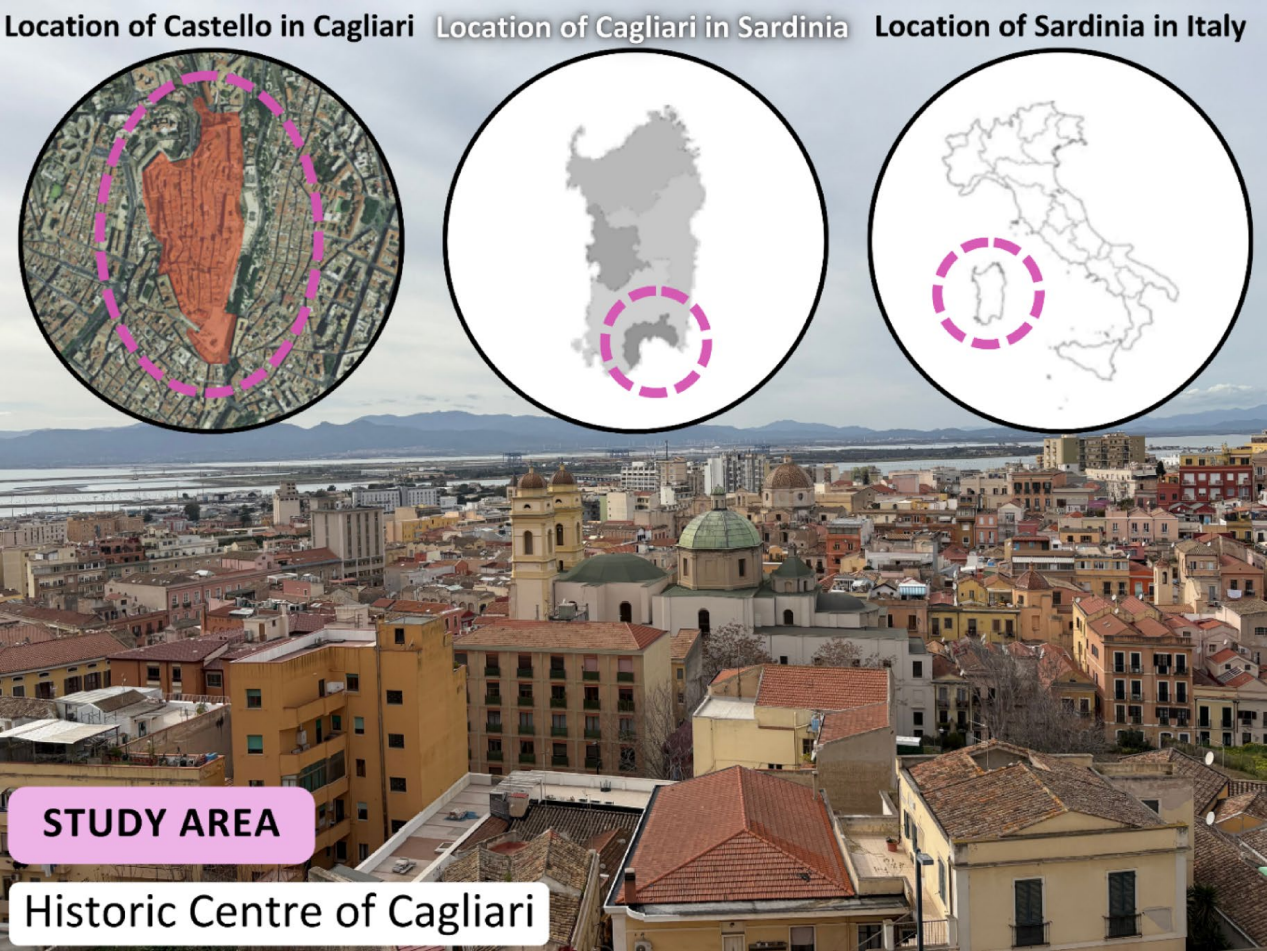
Location maps of the study area in Italy, Sardinia, Cagliari, and Castello neighbourhood in the historic centre of Cagliari. The base map and all images in this figure were created and captured by the corresponding author. The photographs were taken during fieldwork in 2025 and post-edited in Adobe Photoshop CS5.1 (https://www.adobe.com/products/photoshop.html).

This study focuses on the Castello neighbourhood, located in the historic centre of Cagliari, the capital city of the island of Sardinia, Italy (Fig. 2). The historic Castello district, Cagliari’s oldest and most iconic area, presents a compelling case study for exploring adaptable transformation of social spaces. This vibrant neighbourhood, a significant cultural and economic cornerstone of Sardinia with a rich history and lively urban atmosphere, is characterised by its unique medieval architecture, heritage sites, and contemporary urban challenges. Castello’s high historical value, stemming from its 13th-century origins as a fortified medieval district with narrow streets, imposing walls, and notable landmarks like the Cagliari Cathedral and the Bastion of Saint Remy, makes it a prime site for understanding how heritage preservation can be balanced with present-day social requirements.

In addition, as Cagliari’s main tourist centre, attracting both locals and international visitors drawn to its cultural attractions and panoramic views, Castello faces challenges regarding the availability and usability of social spaces. The limited areas for social and recreational activities highlight the need for improved community interaction facilities^58^. Consequently, Castello’s historical significance, coupled with its tourism focus and the scarcity of adaptable social spaces, makes it an ideal location to investigate versatile strategies for converting underutilised areas into thriving public spaces. By concentrating on Castello, this research seeks to address the adaptive reuse of historic sites, the incorporation of social spaces within tourist hotspots, and the role of planning strategies in enabling these changes.

The selection of Castello as a case study was therefore grounded in its dual significance: first, as a morphologically complex historic district facing spatial fragmentation and residual land-use patterns; and second, as a high-density tourist and cultural hub where public space provision is increasingly contested. These conditions offer a fertile ground for testing adaptive reuse strategies. The combined application of Space Syntax, GIS, and qualitative assessment was chosen specifically for its ability to link spatial configuration with land-use dynamics and experiential qualities. This integrative method allows for both objective analysis and context-sensitive evaluation of underutilised urban fragments, supporting replicable planning strategies in similarly constrained environments.

## Results

**Fig. 3:**
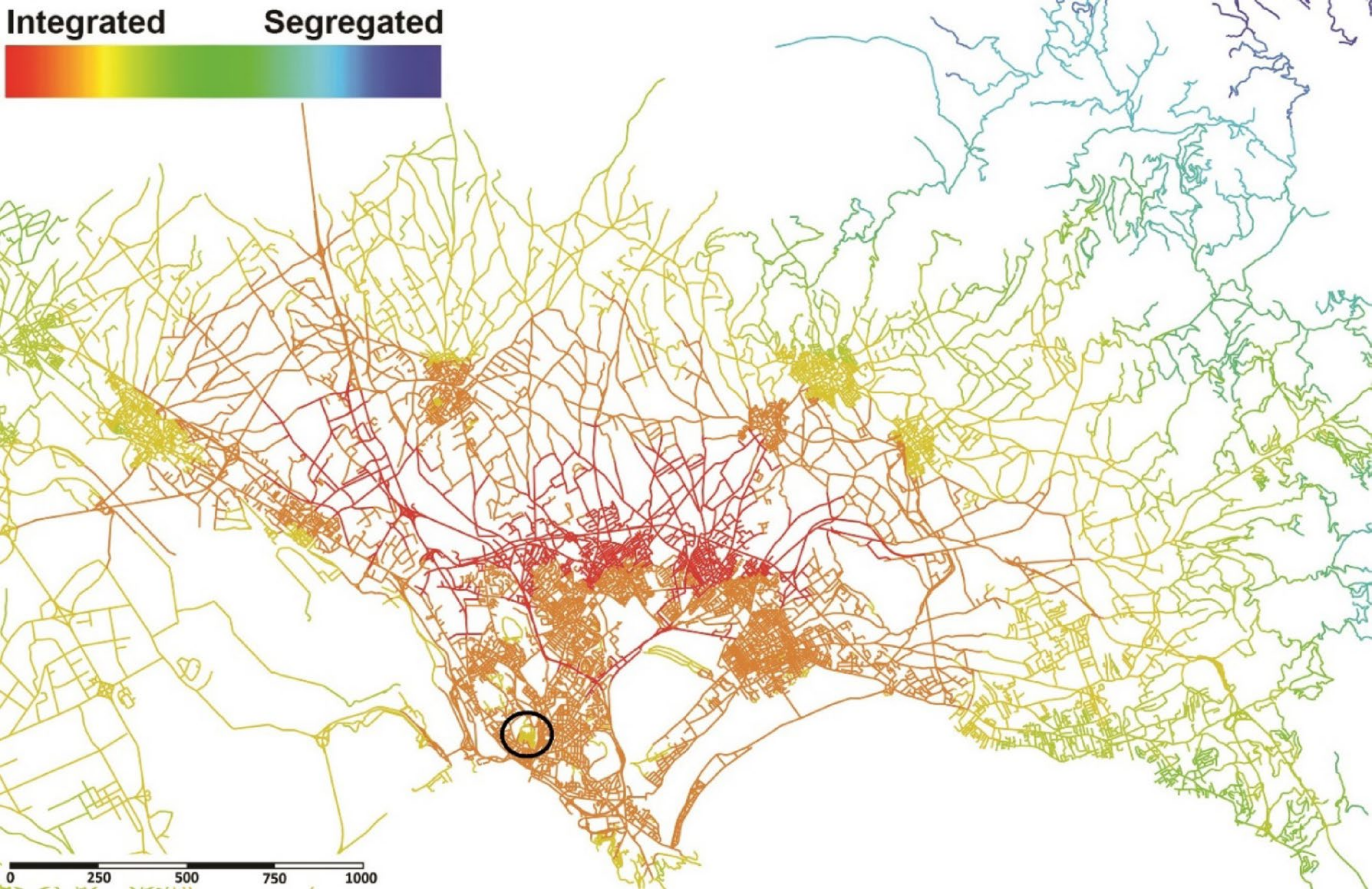
Global integration (HH) analysis performed on a segment map of Cagliari: The target area shows lower integration values (yellow), indicating weaker integration compared to its surrounding areas, which display higher integration values (orange). The analysis was generated by the corresponding author using UCL Depthmap version 10.14.00b (https://github.com/SpaceGroupUCL/Depthmap) and post-edited in Adobe Photoshop CS5.1 (https://www.adobe.com/products/photoshop.html).

The analysis process commenced with a city-scale spatial analysis using Space Syntax segment maps to assess the configurational accessibility of the urban network. The type of integration analysis applied was global Integration (HH), which evaluates accessibility based on topological distances using the fewest directional changes or turns, rather than metric or angular measurements. The results obtained from this analysis revealed that the target area suffers from poor connectivity to the rest of the city, despite its central location (Fig. 3). The spatial analysis indicates that the study area is predominantly characterised by lower integration values, represented in yellow (ranging from 0.102 to 0.104), in contrast to its immediate surroundings, which reveal higher integration values in orange (ranging from 0.105 to 0.107). This yellow spot within the neighbourhood’s configuration reflects the historic fortification of the Castello district, where limited gates and steep level differences increase topological depth. The current discourse may expand the previous discussions^40,59,60^ implying that these characteristics contribute to distinct accessibility patterns compared to modern cities and that the inherent “walled” nature creates a fundamental spatial segregation between the city and its external environment. These findings suggest that, despite its geographical centrality, the target area remains spatially segregated, which may have implications for limiting pedestrian movement, accessibility, and the establishment of social interactions within its historic fabric. This highlights the need for improvements in this neighbourhood to enhance its sociability, as it suffers from spatial segregation despite being one of the city’s most significant attractions.

**Fig. 4:**
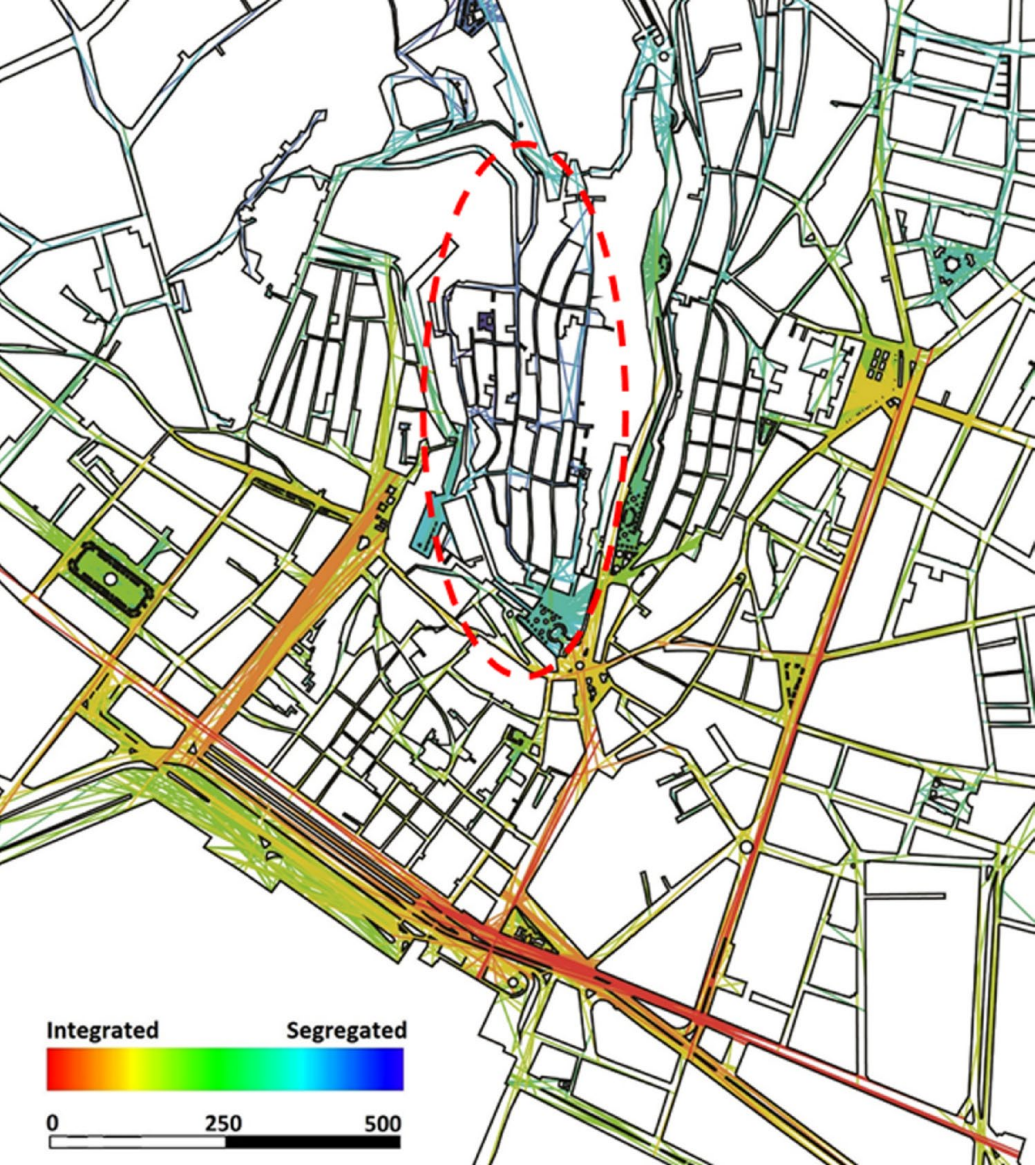
Global Integration (HH) computed on a fewest-line axial map of the Cagliari city-centre street network, highlighting the spatial segregation of the Castello neighbourhood. The analysis was generated by the corresponding author using UCL Depthmap version 10.14.00b (https://github.com/SpaceGroupUCL/Depthmap) and post-edited in Adobe Photoshop CS5.1 (https://www.adobe.com/products/photoshop.html).

The next stage of spatial analysis involved axial line map analysis at the city centre scale to assess potential movement patterns in the vicinity of the neighbourhood. Since socio-behavioural studies typically focus on spaces rather than lines, examining the spatial configuration between buildings is essential for accurately simulating pedestrian movement patterns using axial line maps. The integration graph derived from the axial line analysis confirmed that the spatial configuration of the Castello historic neighbourhood is somewhat segregated from the main streets in its immediate surroundings (Fig. 4). The quantitative integration values for the Castello neighbourhood ranged between 0.86 and 2.01, whereas the integration values of its surrounding streets varied from 2.53 to 3.03, highlighting its relative spatial isolation. These findings further reinforce the notion that despite its historical significance, Castello’s spatial structure limits its permeability, potentially affecting pedestrian flow and social engagement.

The next stage of analysis involved conducting a VGA within the Castello neighbourhood. This analysis examined four key spatial properties within the VGA framework: visual integration, visual connectivity, visual control, and visual clustering coefficient, to comprehensively assess the syntactic and intervisibility characteristics of the area. The visual integration analysis identified Piazza Carlo Alberto (4.85), Piazza Palazzo (4.43), and Piazzetta Maria Lai (4.24) as the three most integrated spaces. Regarding visual connectivity, Bastione di Saint Remy, renowned as the most prominent landmark of the city, emerged as the most visually connected space, with a connectivity value of 1516. This was followed by Piazza Palazzo (1403) and the vicinity of Piazzetta Mercedes Mundula (1266), which were also identified as highly connected spaces.

**Fig. 5:**
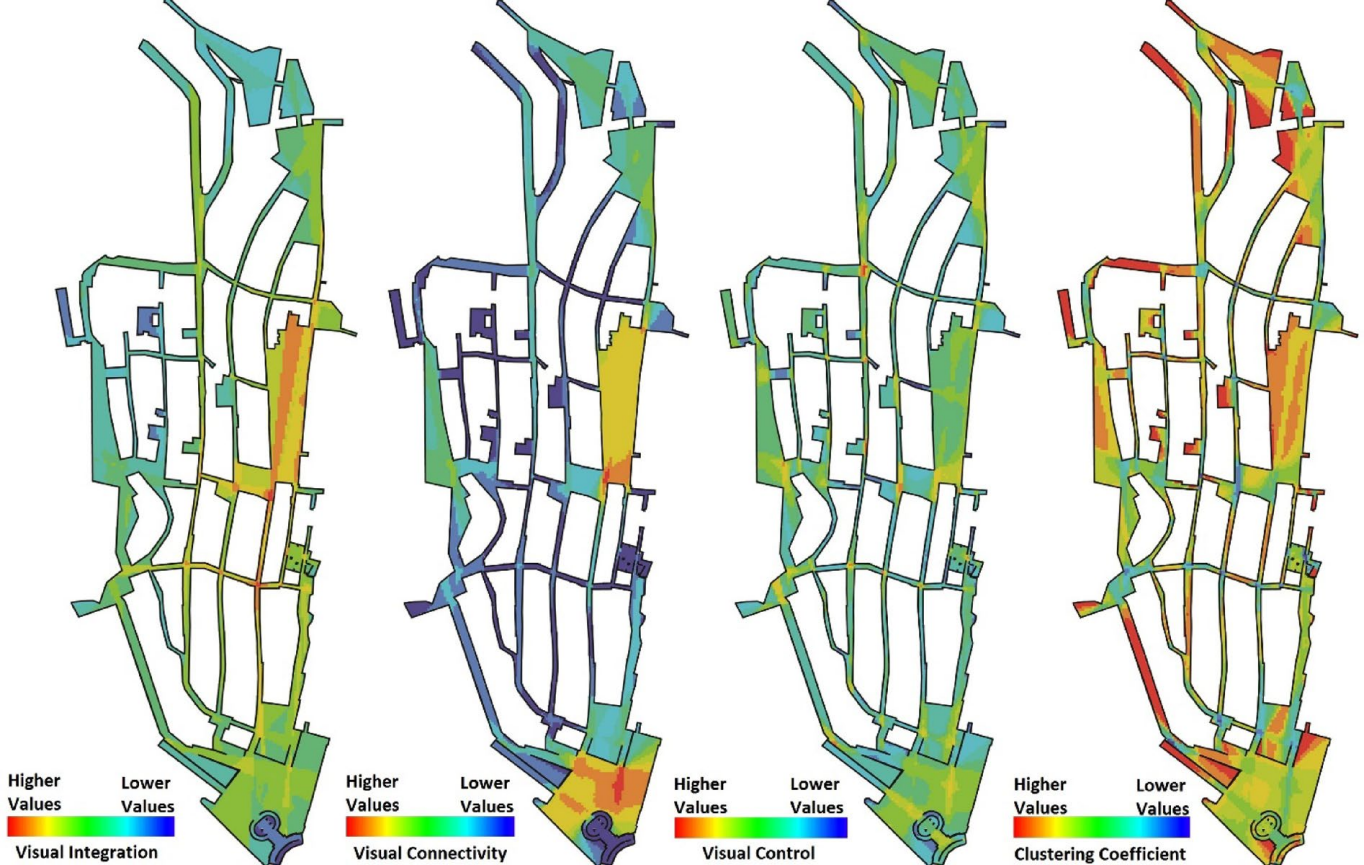
VGA results of the Castello neighbourhood, illustrating integration, connectivity, control, and clustering coefficient values at the neighbourhood scale. The analysis was generated by the corresponding author using UCL Depthmap version 10.14.00b (https://github.com/SpaceGroupUCL/Depthmap) and post-edited in Adobe Photoshop CS5.1 (https://www.adobe.com/products/photoshop.html).

The visual control analysis revealed that the Piazza Carlo Alberto exhibited the highest control value (2.01), indicating its potential influence on movement patterns. Afterward, Piazza Palazzo and Piazza Roberto Coroneo display the highest visual control among potential communal spaces, with values of 1.87 and 183, respectively. Finally, the visual clustering coefficient highlighted certain locations as key pausing spots within the neighbourhood, with Piazza Roberto Coroneo (1.00), Bastione di Saint Remy (0.99), and the landscape viewpoint at Piazzetta Mercedes Mundula (0.98) demonstrating the highest clustering values (Fig. 5). The variations in syntactical values across the identified plazas suggest that while some spaces display strong potential for fostering social interactions due to their high integration and connectivity, others display spatial characteristics that could be leveraged for strategic urban interventions to enhance sociability.

**Fig. 6:**
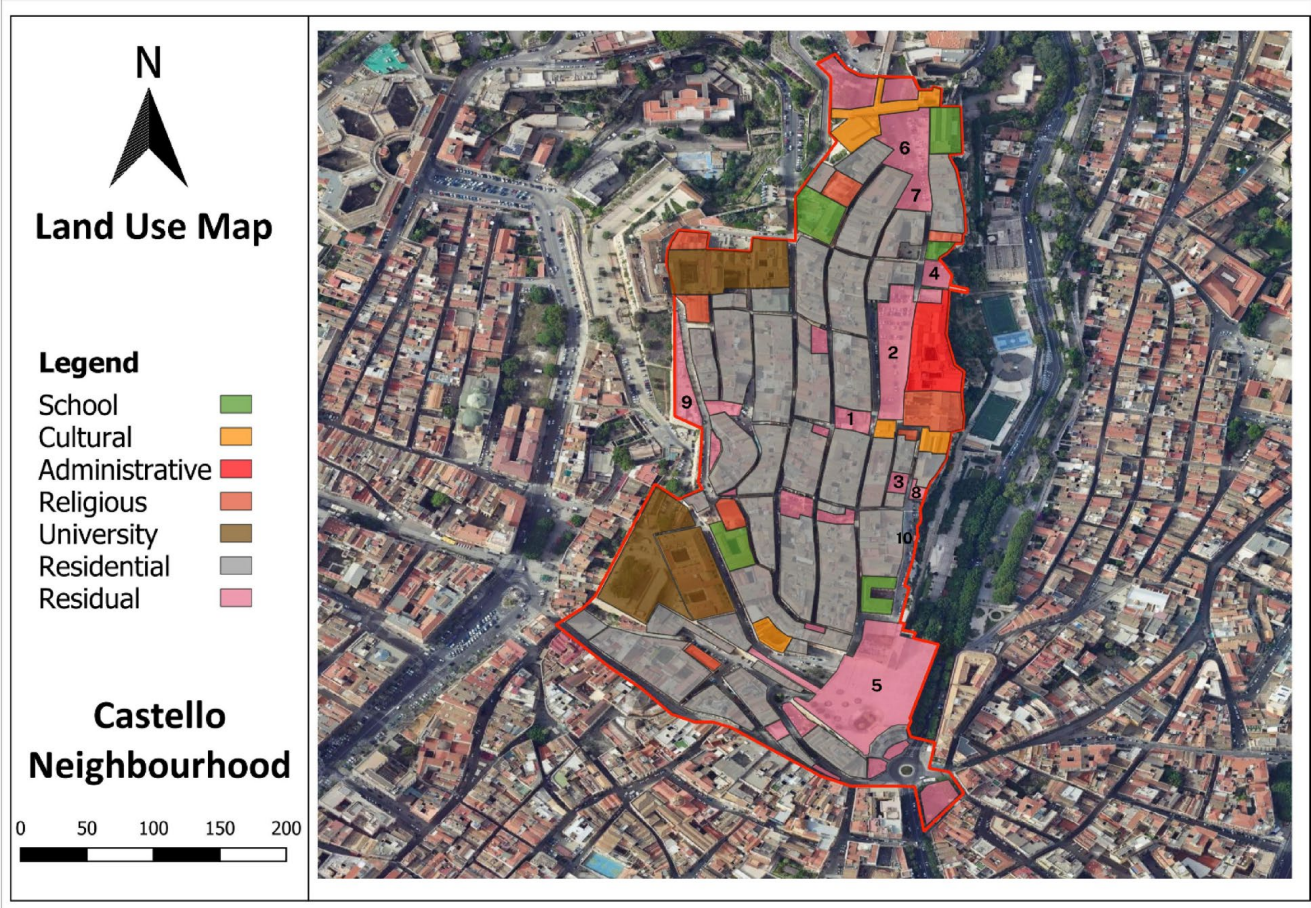
Land use classification of the Castello neighbourhood, highlighting residual spaces as potential areas for urban intervention. This map was created by the corresponding author using QGIS version 3.16 (https://qgis.org/).

Following the syntactical analysis, a land use classification was conducted to identify potential residual spaces within the Castello neighbourhood. The land use map was developed using QGIS, categorising various functions such as residential, administrative, cultural, educational, religious, and university buildings, alongside residual spaces (Fig. 6). The analysis reveals that residential areas dominate the neighbourhood, interspersed with administrative and cultural functions, primarily located along the main streets. Notably, residual spaces, marked in pink, are scattered throughout the area, with a concentration along the eastern and southern peripheries. These spaces often appear adjacent to historical buildings, within underutilised or fragmented urban pockets. Their distribution suggests potential opportunities for urban interventions aimed at enhancing social interactions and public engagement. The proximity of these residual spaces to key public and cultural landmarks such as *Bastione di Saint Remy* and *Santa Maria Assunta e Santa Cecilia Cathedral*, further supports their adaptability for conversion into inclusive social spaces. This spatial assessment provides critical insights for strategic urban design solutions that could reintegrate these spaces into the neighbourhood’s social fabric.

The syntactical analysis of residual spaces within the Castello neighbourhood reveals distinct trends across the four key metrics consisting of integration, connectivity, control, and clustering coefficient, highlighting their spatial potential (Table 1). The integration analysis indicates that Piazza Carlo Alberto (4.85), and Piazza Palazzo (4.43) possess the highest integration values, suggesting their strong potential to attract pedestrian crowds and the possibility of social encounters. Connectivity values show Bastione di Saint Remy (1516) as the most visually connected space, followed by Piazza Palazzo (1403) and Piazzetta Mercedes Mundula (1266), reinforcing their prominence in the spatial accessibility. In terms of visual control, Piazza Carlo Alberto (2.01), and Piazza Palazzo (1.87) indicate higher values, signifying their influential surveillance over movement distribution. Clustering coefficient values remain consistently high in Bastione di Saint Remy (0.99) and Piazza Roberto Coroneo (1.00), indicating strong localised visibility, potential hotspots for social interactions, and establishment of static social behaviours (Table 1). To ensure spatial clarity, the corresponding locations of the ten identified residual spaces are labelled on the land use map in Fig. 6 using numerical references matching those listed in (Table 1). Overall, Piazza Palazzo, Piazza Carlo Alberto, and Bastione di Saint Remy emerge as the most syntactically significant residual spaces, suggesting their suitability for urban interventions aimed at enhancing sociability and spatial integration. These findings align with the land use analysis for identifying residual spaces, confirming their strategic role in reintegrating underutilised spaces into the urban fabric.

**Table 1:** Numerical data obtained from different metrics of VGA across potential residual lands.

Locations	Integration	Connectivity	Control	Clustering coefficient
1. Piazza Carlo Alberto	4.85	1373	2.01	0.85
2. Piazza Palazzo	4.43	1403	1.87	0.97
3. Piazzetta Maria Lai	4.24	343	1.83	0.83
4. Piazzetta Mercedes Mundula	4.29	1266	1.70	0.98
5. Bastione di Saint Remy	4.07	1516	1.79	0.99
6. Piazza dell Indipendeza	3.47	822	1.63	0.98
7. Piazzetta Mafalda di Savoia	3.94	916	1.45	0.98
8. Piazza Roberto Coroneo	4.05	293	1.83	1.00
9. Bastione di Santa Croce	3.34	855	1.87	0.91
10. Via del Fossario	3.03	212	0.57	0.98

**Fig. 7:**
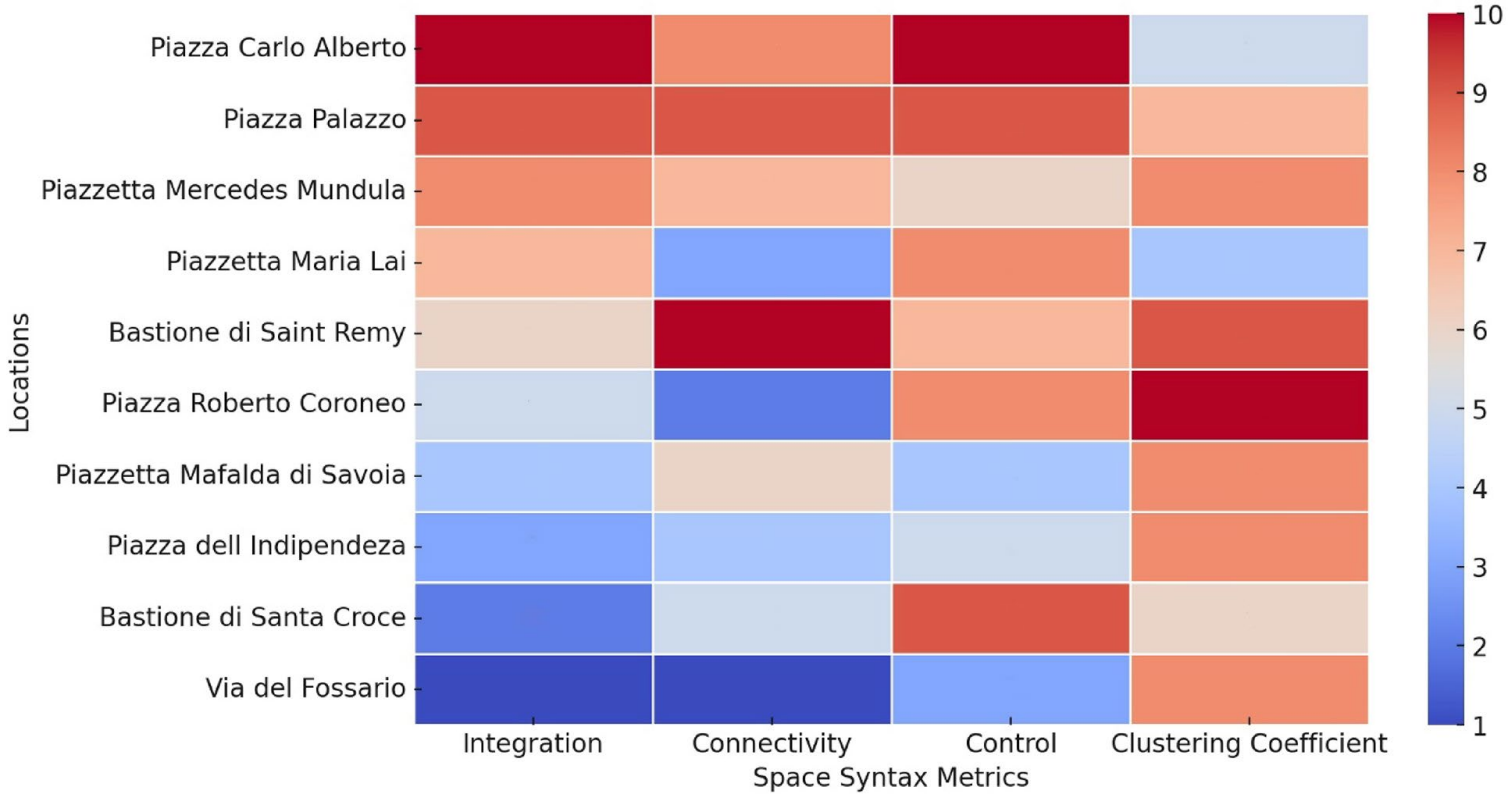
Heatmap of space syntax metrics obtained from VGA and their associated credits based on their spatial values.

The heatmap provides a comparative ranking of urban residual spaces based on their syntactical values, suggesting their potential for encouraging social interactions (Fig. 7). According to the aggregated credit system, Piazza Palazzo (34 credits), Piazza Carlo Alberto (33 credits), and Bastione di Saint Remy (32 credits) emerge as the most significant locations for social activities. Piazza Palazzo, with consistently high values across Integration, Connectivity, and Control, demonstrates strong spatial influence, making it a prime candidate for urban social interactions. Piazza Carlo Alberto, ranking second, reveals outstanding Integration and Control values, suggesting its critical role in overall pedestrian movement. Bastione di Saint Remy, closely following, stands out due to its highest Connectivity value and strong Clustering Coefficient, indicating its potential as a locally cohesive space for interaction. Additionally, Piazzetta Mercedes Mundula (29 credits) and Piazza Roberto Coroneo (25 credits) present considerable socialisation potential due to their balanced syntactical values. These findings affirm a strong correlation between the spatial configuration of these locations and their capacity to facilitate social encounters, positioning them as key urban nodes for enhancing public life. The vertical axis of this heatmap does not represent a continuous numerical scale; instead, the credit values for each syntactic metric (ranging from 1 to 10) are used as a ranking-based scoring system to visually compare spatial performance across different locations.

**Fig. 8:**
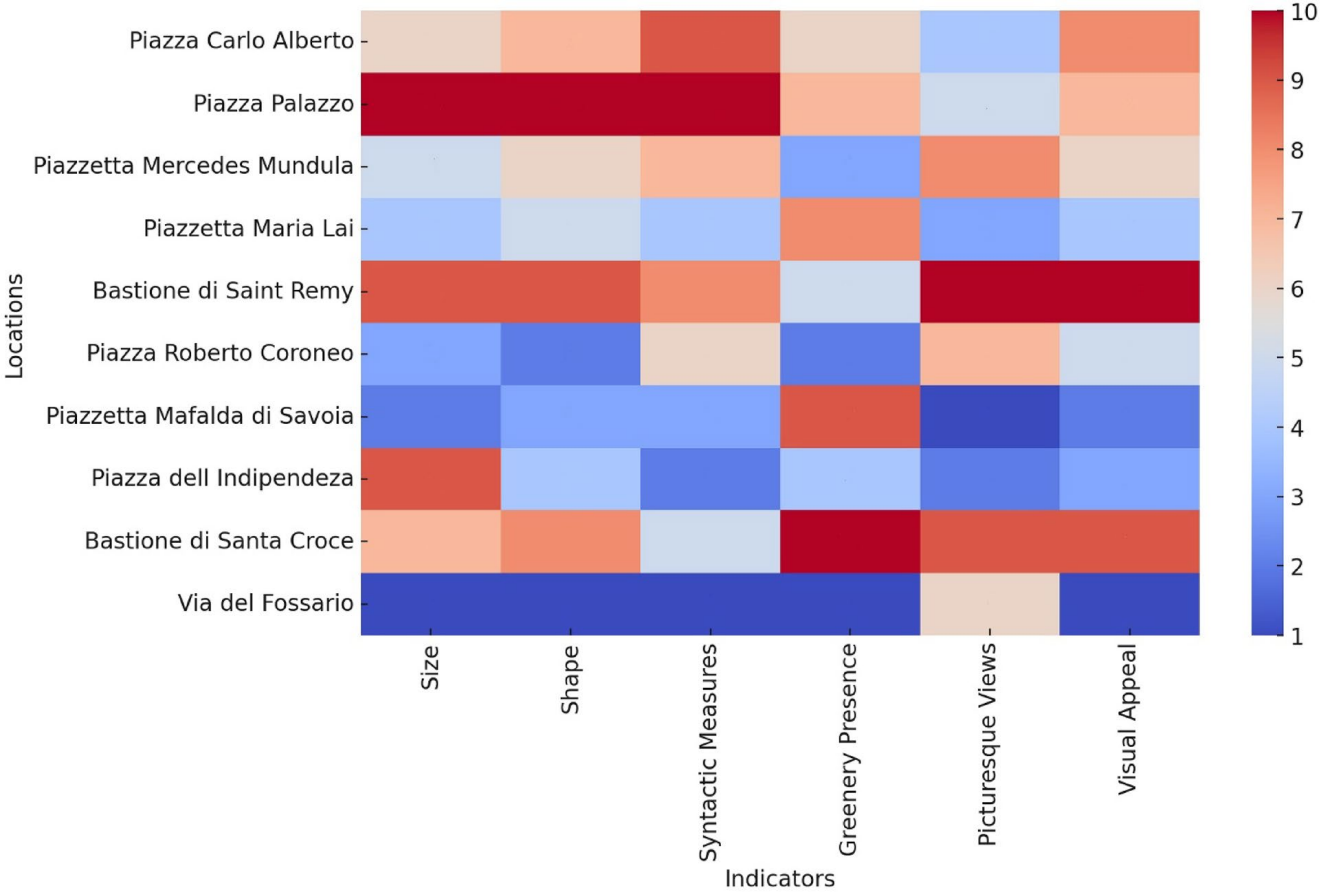
Heatmap illustrating the distribution of coloured spectrum values for various spatial and aesthetic indicators across different locations.

In the next step, other aspects of spatial quality assessment, including physical characteristics, spatial qualities, landscape elements, and aesthetic qualities, were recorded and documented through field investigations. Figure 8 visually represents the numerical values of different indicators across various locations using the heatmap. The color gradient, ranging from blue (lowest values) to red (highest values), allows for an intuitive interpretation of the data distribution. Notably, locations such as Piazza Palazzo and Bastione di Saint Remy display consistently high values across all indicators, signifying well-balanced spatial and aesthetic qualities. Conversely, via del Fossario and Piazzetta Mafalda di Savoia register the lowest scores, particularly in syntactic measures and aesthetic appeal, indicating potential areas for improvement. The heatmap highlights strong greenery presence in Bastione di Santa Croce and Piazzetta Mafalda di Savoia, while Piazza Carlo Alberto and Bastione di Saint Remy score highly in visual appeal. This comparative analysis aids in identifying locations with superior spatial and aesthetic attributes while pinpointing areas needing further development for a more balanced urban experience (Table 2). It should be noted that the vertical scale in this heatmap reflects the scores (1 to 10) derived from expert evaluations and field observations of qualitative indicators, and not raw or continuous measurement values. These scores were used to allow visual and comparative analysis of spatial and aesthetic features.

**Table 2:** The numerical values for various physical, spatial, landscape, and aesthetic qualities across different locations.

Locations	Physical characteristics		Spatial qualities	Landscape elements		Aesthetic qualities
	Size	Shape	Syntactic measures	Greenery presence	Picturesque views	Visual appeal
Piazza Carlo Alberto	6	7	9	6	4	8
Piazza Palazzo	10	10	10	7	5	7
Piazzetta Mercedes Mundula	5	6	7	3	8	6
Piazzetta Maria Lai	4	5	4	8	3	4
Bastione di Saint Remy	9	9	8	5	10	10
Piazza Roberto Coroneo	3	2	6	2	7	5
Piazzetta Mafalda di Savoia	2	3	3	9	1	2
Piazza dell Indipendeza	9	4	2	4	2	3
Bastione di Santa Croce	7	8	5	10	9	9
Via del Fossario	1	1	1	1	6	1

**Fig. 9:**
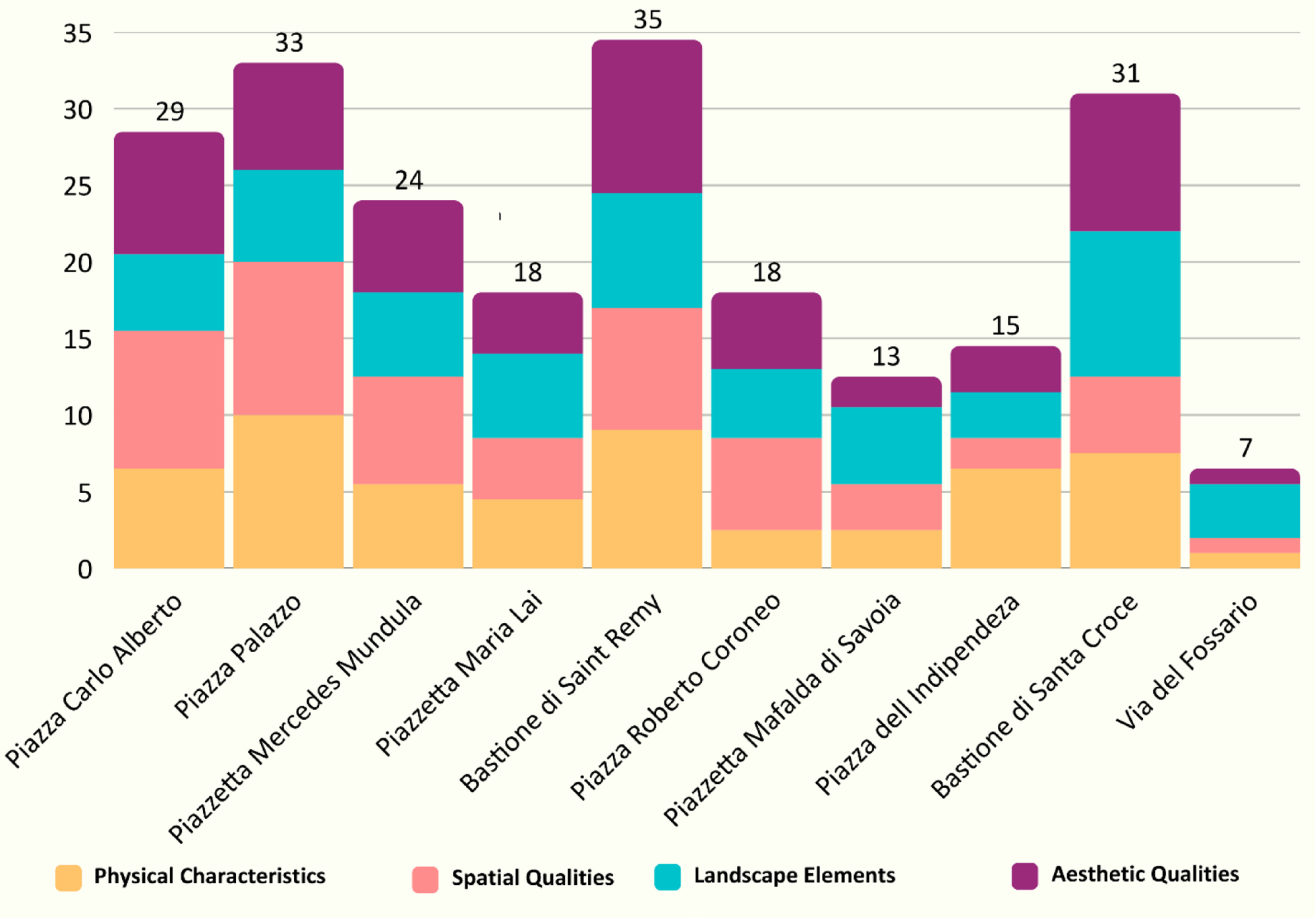
Comparative assessment of urban residual spaces based on physical characteristics, spatial qualities, landscape elements, and aesthetic qualities, indicating their potential for conversion into social spaces.

Ultimately, the stacked bar chart illustrates the comparative assessment of various urban residual spaces based on four key attributes: physical characteristics, spatial qualities, landscape elements, and aesthetic qualities (Fig. 9). Each of these attributes contributes to the overall potential of a space for conversion into a socially vibrant area. The height of the stacked bars represents the total score of each space, indicating their relative importance and suitability for urban transformation. Notably, Bastione di Saint Remy, Piazza Palazzo, Bastione di Santa Croce, and Piazza Carlo Alberto emerge as the most significant spaces, scoring the highest across multiple metrics. These spaces indicate a balanced composition of physical, spatial, landscape, and aesthetic attributes, making them strong candidates for social space conversion. In contrast, via del Fossario ranks the lowest, suggesting limited potential due to lower scores across all four categories. The distribution of different attributes within each space further underscores their strengths and weaknesses, providing insightful visions into urban renewal priorities. This analysis aids in identifying key intervention areas where strategic improvements can enhance public engagement, walkability, and social interactions.

## Discussion

The obtained results and adopted unique procedures associated with this study revealed innovative sequential methods for addressing the primary aims of this study. The findings suggest that the use of such exploratory methods may help clarify certain ambiguous aspects of identifying and applying adaptable solutions to convert residual street spaces into vibrant social hubs. Rather than presenting a universally generalisable framework, this approach is intended for adaptation in cities with similar morphological and spatial conditions as the case study site. In essence, the study proposes a versatile and adaptable methodological pathway for addressing the challenges and opportunities presented by urban regeneration of residual street spaces in morphologically similar cities worldwide, both domestically and internationally.

While the current study primarily approached residual spaces from a spatial-analytical perspective, we acknowledge that residuality is not solely a morphological or functional condition—it is also shaped by socio-political, economic, and governance-related dynamics. Residual spaces may emerge due to exclusionary planning practices, historic fragmentation, neglected infrastructural remnants, or privatised urban redevelopment. Although the scope of this study did not develop a detailed typological or socio-political classification of residual street spaces, it conceptually frames them as urban voids that are under-programmed and insufficiently integrated into the everyday life of the city, despite their formal designation as plazas or urban fragments. The methodological framework presented here identifies such spaces based on their physical availability, spatial centrality, and lack of functional characteristics, rather than reducing residuality to visual emptiness alone. We recognise that attributes such as land tenure, perceived safety, informal usage, and community engagement significantly affect their social potential; however, these contextual layers were beyond the empirical scope of this study and are proposed as future areas of exploration.

Importantly, the identification of residual spaces in this study was not based solely on low integration or syntactic segregation. Several selected spaces, such as Piazza Palazzo and Piazza Carlo Alberto, demonstrated relatively high configurational values, yet still exhibited clear signs of underutilisation and social underperformance due to a lack of programmatic activation, design amenities, or governance attention. This reinforces the central argument that residuality is not equivalent to syntactic marginality, but rather a composite condition revealed through the misalignment between spatial potential and actual usage.

The spatial analysis revealed that spaces such as Piazza Palazzo, Piazza Carlo Alberto, and Bastione di Saint Remy displayed consistently high integration, connectivity, and control values, supporting their potential as future social hubs. This aligns with previous research^61,62^ which highlighted that spatial centrality and intervisibility are critical predictors of successful social space reactivation. However, unlike prior studies that primarily viewed residual land through ecological or aesthetic lenses^63,64^ our findings indicate that some seemingly ‘active’ spaces—despite their historical or architectural value—remain underutilised in terms of social interaction due to lacking urban design interventions. For example, the Bastione di Saint Remy, while architecturally prominent, still contains vast unprogrammed surfaces that reduce its sociability. The employed method reveals how syntactic values and spatial fragmentation affect local accessibility, offering a fine-grained, spatialised explanation of residuality that complements broader ecological or tactical urbanism perspectives.

This study’s findings also complement recent work that foregrounds pedestrian perception and narrative-based assessments, which used storytelling to evaluate how spatial changes in Cairo’s residential streets affected everyday pedestrian scenarios^65^. While their approach is more qualitative and perception-driven, our spatial methodology converges with their conclusion that socio-spatial alterations of land uses without grounded assessment criteria can diminish walkability and social safety of pedestrians in urban public spaces. Similarly, yet another research which integrates qualitative and quantitative walkability indicators to promote resilient urban design^66^ shares conceptual alignment with our study. Both studies reinforce that successful street activation hinges not only on spatial accessibility but also on physical facilities, perceptual coherence, and inclusivity, themes our findings support through the combined use of Space Syntax, land use mapping, and field-based qualitative assessments. Together, these perspectives highlight the growing importance of integrating pedestrian needs, configurational analysis, and social context in urban regeneration strategies.

While prior studies have explored the potential of vacant and residual urban spaces, ranging from neglected parking lots^19,21^ to voids beneath elevated highways^6^ their focus has largely been on temporary applications or enhancing ecological and social resilience. In contrast, this study proposes a context-specific methodological framework for spatial prioritisation by strategically combining Space Syntax metrics with GIS-based land use mapping and on-site investigations. This allows for the identification and prioritisation of residual spaces based on quantified spatial qualities like integration, connectivity, control, and clustering coefficient, moving beyond the advocacy for flexible reuse^26,27^ or purely ecological and social value enhancement^23,29^ found in earlier work. Rather than claiming methodological exclusivity, this study contributes a structured application of established tools in a historically layered context, enabling governance-led, data-informed decisions for the adaptive reuse of historic urban voids. It thus offers a strategic pathway for converting underutilised street spaces into social nodes and enriches the urban regeneration discourse through spatially-informed and governance-oriented planning.

Although previous research has thoroughly explored the ecological benefits^22,23^classification^57^ temporary cultural uses^25^ and placemaking implications^32^ of residual spaces, the systematic transformation of these spaces into socially functional urban places has received less direct attention. What distinguishes this study is its explicit focus on converting residual street spaces into social hubs within historic urban contexts. By combining spatial configuration analysis, land use data, and qualitative site assessments, this research addresses a critical gap: the lack of structured, spatially-informed strategies for enhancing the sociability of neglected urban fragments. While prior studies have called for adaptability, flexibility and inclusivity^24,31,67^, this study provides a replicable spatial methodology that directly links the physical characteristics of urban voids to their potential for cultivating social interaction. This distinctive approach offers both a theoretical contribution and a practical framework for reactivating residual spaces as inclusive, everyday social destinations.

This study offers both theoretical and practical implications to the fields of urban design, spatial planning, and adaptive urban governance. Theoretically, it advances the discourse on residual space by introducing a spatially grounded framework that integrates Space Syntax metrics with land use classification and qualitative field analysis, enabling a deeper understanding of how spatial configuration influences social behaviour. It bridges the gap between abstract spatial theories and real-world social needs by demonstrating how configurational properties can inform the selection and transformation of underutilised spaces. Practically, this research provides a replicable methodology for municipalities, urban planners, urban governors, and policymakers seeking to revitalise leftover urban areas into vibrant, socially inclusive spaces, particularly within historic or high-density contexts where land is scarce. By prioritising spaces based on quantifiable social potential, the study facilitates evidence-based decision-making and supports the development of adaptable governance strategies that balance spatial performance, heritage conservation, and community needs. Practically, the prioritised residual spaces identified through this methodology offer tangible candidates for targeted urban interventions, such as placemaking initiatives, micro-programming, and low-cost design modifications to enhance spatial usage. Local authorities and urban design agencies can utilise the spatial rankings and visibility maps as decision-support tools for prioritising public investment or community activation programs. The framework thus holds direct utility for operationalising evidence-based, small-scale urban regeneration within constrained historic contexts.

While this study provides a solid methodological framework for identifying and prioritising residual street spaces for social transformation, it is not without limitations. First, the research lacks empirical user-based data such as surveys, interviews, or real-time behavioural tracking, which could further validate the social potential of the selected spaces. Additionally, the analysis did not incorporate design interventions or visualisations to test how spatial modifications might influence actual usage. The study also focused solely on a historic neighbourhood, limiting its generalisability across recently developed contexts, where spatial dynamics and governance structures may differ significantly. Furthermore, while the framework offers a governance-oriented perspective, it stops short of engaging with specific urban governance models or policy mechanisms tailored to residual street spaces.

For future research, it would be valuable to conduct comparative studies across different urban fabrics, examining how residual spaces function in both historic and newly planned environments. This seven-phase framework, detailed in Fig. 1, provides a clear roadmap that can be replicated or adapted for cities with similar morphological or spatial challenges. In particular, future applications of this methodology could be explored in cities with comparable urban morphologies and topographical constraints. Examples include historic centres in cities such as Naples, Genoa, and Palermo in Italy, or internationally in Lisbon (Portugal), Istanbul (Turkey), and Valparaíso (Chile). These sites share structural similarities with Cagliari and could benefit from similar adaptive strategies. Incorporating walkability indices, stakeholder interviews, and community-based participatory methods could deepen understanding of local needs and nurture more inclusive design strategies. Scholars might also explore the implementation of adaptive governance models, or multifunctional land-use scenarios to activate residual spaces more holistically. Finally, future studies should aim to include quantitative empirical data on the social, economic, and health impacts of reactivated residual spaces, thereby reinforcing the broader value of spatial interventions in improving urban quality of life.

## Conclusion

This study aimed to explore how residual street spaces, particularly within historic urban contexts, can be strategically transformed into vibrant social spaces. The research highlights the critical importance of identifying and activating underutilised urban fragments as a means to enhance social interaction, social inclusivity, and spatial equity. The outcome of this study establishes that spatial configuration plays a fundamental role in shaping social functionality, and when combined with qualitative assessments, it can guide adaptive reuse strategies in a targeted and evidence-based manner. The originality of this work lies in its multi-scalar, sequential nature, and data-driven framework that bridges spatial analysis and urban regeneration, moving beyond the conceptual exploration of residual spaces to offer a practical methodology for social transformation.

While grounded in the context of an Italian city, the proposed framework is transferable to cities worldwide, particularly those with complex historic fabrics or fragmented urban morphologies. In conclusion, this research repositions overlooked urban spaces as powerful assets in the pursuit of socially inclusive and spatially just cities. However, it is important to acknowledge several limitations of the study: the absence of user-based empirical data, the lack of design simulations for future spatial interventions, and the geographic focus on a single historic neighbourhood, which may limit broader generalisation. Future studies are encouraged to address these aspects and expand upon the proposed framework across diverse urban contexts.

## Data Availability

The data that support the findings of this study are available from the authors upon reasonable request. Restrictions may apply to the availability of the data due to privacy and ethical considerations.
